# Unlocking the Carbyne-Enriched Nanocoating Sensitivity to Volatile Organic Vapors with Plasma-Driven Deposition onto Bulk Micromachined Silicon Membranes

**DOI:** 10.3390/nano12122066

**Published:** 2022-06-15

**Authors:** Mariya Aleksandrova, Georgi Kolev, Georgi Dobrikov, Andrey Brigadin, Alexander Lukin

**Affiliations:** 1 Department of Microelectronics, Technical University of Sofia, 1000 Sofia, Bulgaria; georgi_klv@abv.bg (G.K.); georgi_hd@tu-sofia.bg (G.D.); 2 Swissimpianti Sagl, 6828 Balerna, Switzerland; info@swissimpianti.ch; 3 Western-Caucasus Research Center, 352808 Tuapse, Russia; lukin@wcrc.ru

**Keywords:** bulk micromachined silicon substrates, microfabrication technology, carbyne-enriched nanocoatings, ion-assisted pulse-plasma deposition, volatile organic compounds

## Abstract

Due to the unique combination of physicochemical and structural properties of carbyne-enriched nanocoatings, they can be used for the development of high-end electronic devices. We propose using it for the development of sensor platforms based on silicon bulk micromachined membranes that serve as a part of microcapacitors with flexible electrodes, with various sizes and topologies. The carbyne-enriched nanocoating was grown using the ion-assisted pulse-plasma deposition method in the form of 2D-ordered linear-chain carbon with interchain spacing in the range of approximately 4.8–5.03 Å. The main characteristics of the fabricated sensors, such as dynamic range, sensitivity, linearity, response, and recovery times, were measured as a function of the ethanol concentration and compared for the different sizes of the micromembranes and for the different surface states, such as patterned and non-patterned. The obtained results are the first step in the further optimization of these sensor platforms to reach more precise detection of volatile organic compounds for the needs of the healthcare, air monitoring, and other relevant fields of human health.

## 1. Introduction

Carbon-containing nanomaterials are essential for gas sensor devices, as they have suitable physical and chemical properties that boost the detection of particles and pollutants dispersed in the liquid or gas phase. The application of carbyne-enriched nanocoatings for sensing applications is a relatively unexplored field with great potential [1]. In this material, the ordered array of one-dimensional carbon chains packed parallel to one another in hexagonal structures is oriented perpendicular to the substrate surface. In carbon atom chains, each carbon atom is connected to the two nearest neighbors by sp-1 bonds. Two-dimensionally ordered linear-chain carbon nanomatrices represent a multi-cavity nanostructure, containing vacant functional nanocavities, available for assembling by atom clusters of various chemical elements. Such vacant functional nanocavities can be considered analogues of cavities in carbon nanotubes, for which studies have been performed previously [2]. 

The concerns that have been raised about environmental issues have accelerated research efforts into the development of chemical sensors, and thus several volatile organic compounds’ detection techniques have been elaborated with the potential to be commercially implemented. Since sensors should be characterized as having a high sensitivity, accuracy in the ppm range, and second range response for target chemical analytes, an appropriate option for a cost-effective and miniature devices is the use of micromachining as a fabrication technology. Micromachining is compatible with the microfabrication flow of the electronic chips serving as power conditioning systems for the further processing of sensor signals [3,4]. Among these, cantilevers and capacitive- and acoustic-wave-based microsensors have exhibited a great potential for integration with electronics and the realization of complete products for market needs [5]. 

In the case of cantilever mass-sensitive sensors, there are two basic constituents: adsorbing layers, such as carbon-based coatings and nanostructures, and a beam with a specific geometrical dimensions. The main resonant frequency of the device is strongly dependent on the additional mass of the cantilever beam due to the number of adsorbed molecules. As a result, the device frequency shifts toward lower frequencies, and this response is reversible once the gas or vapor molecules are desorbed. This principle of detection is characterized by a high resolution of nanograms that can be registered. However, the stimulation of mechanical motion (deflection) of these structures requires non-conventional assembling, mounting, and packaging processes, unlike the planar devices with no movable parts [6]. 

The principle of surface acoustic wave (SAW) sensors has been also applied, where the combination of input and output transducers serve as a time-delay line or an electrical signal phase shifter. Its operation can be ascribed to a certain amount of analyte molecules absorbed on the layer, distributing the wave [7]. However, this principle is well developed for the combination of inorganic sensing layers and metal electrodes. When organic sensing layers contact metal films, there is a non-Ohmic contact, resulting in electrical losses due to the voltage drop at the junctions. The improvement of the sensor characteristics can be achieved if materials that are in contact are similar in nature—for example, the organic/organic interface. For now, organic interdigital electrodes have not been developed, including as a bi-layer system with organic gas sensing layer.

Capacitive sensors have many advantages over other types of sensors because of their negligible temperature dependence, the possibility for linear response with a proper design, and ultra-low power requirements [8,9]. Capacitive gas/vapor sensors also have specific advantages over resistive devices. Resistive sensors rely on the transfer of charges between the sensing layer and contact electrodes that is susceptible to flicker (1/f) noise and incomplete recovery of charges. However, the detection mechanism in a capacitive sensor is principally dominated by tracking the change in the dielectric properties of the sensing layer and does not involve any transportation of charges. Thus, it provides more sensitivity and reliability for chemical detection. Additionally, capacitive sensors consume no static power and thus are very much in demand in energy-constrained environments. Their miniaturized size, the minimal power requirements, the integration of many individual capacitors in a small footprint, and the availability of commercial readout electronics for the accurate measurement of small capacitance changes make them a promising transduction platform for gas/vapor sensing implementations at the Point of Need [10]. Capacitive sensors have already been integrated with several promising sensing materials and have demonstrated the stable and reversible sensing of many Volatile Organic Compounds (VOCs) such as ethanol, methanol, and formic acid. Recently, a novel gas sensor, based upon vapor-induced capacitance modulation of chemically functionalized porous graphene oxide (pGO), has been developed [11]. Excellent vapor sensing properties have been demonstrated, specifically, extremely high sensitivity, wide dynamic range, and rapid response and recovery times. The problems related to these principles of operation such as performance affected by the ambient conditions (temperature, humidity) through the variations in the sensing layer permittivity can be overcome with the chemical synthesis of the adsorbing layer and control over its chemical reactivity. The shape and size of the membrane-based movable electrode of the microcapacitor are responsible for the dynamic range, detection rate, and linearity; therefore, they can be varied to tune these properties according to custom requirements. 

In this paper, an ethanol sensing device was fabricated and studied based on a microcapacitor with bulk micromachined silicon membrane as an electrode and coating from the new carbyne-enriched material grown by ion-assisted pulse-plasma deposition technique on different membrane sizes. Ethanol is a flammable, colorless, volatile organic compound (VOC), with application in the food, medicine, pharmacology, and microelectronic industries as a precursor for other organic compounds. In this regard, there is a need for its quantitative measurement with different levels of precision according to the application. However, the detection ability in the of range 50–500 ppm is beneficial for all kinds of practical applications. The main characteristics of the sensing device, such as dynamic range, linearity, sensitivity, and response and recovery time, were measured as a function of the ethanol concentration. The carbynes are emerging as a new class of strong but light materials that could be the next revolution of carbon in material science for electronics. Carbyne films have a high surface area, which is an excellent precondition for broad dynamic range for the target analytes. However, a very limited amount of information exists on the practical application of carbynes in general and in particular for the detection of VOCs in the gas phase. To the authors’ knowledge, this is the first demonstration of novel coating implemented in a practically useful membrane-based sensing structure and the first confirmation of the ability of a carbyne-enriched MEMS capacitive sensor to detect ethanol vapors. The results could be useful for the optimization of this sensor architecture or for the comparison of the results with other sensing platforms, thus finding the most suitable detection principle for the precise detection of VOCs depending on the application (in medicine, healthcare, food or drink industry, air composition monitoring, microfabrication, etc.).

## 2. Materials and Methods

Silicon wafers with crystallographic orientation <100> were preliminary cleaned in 10% hydrofluoric acid from the native SiO_2_. Cleaned wafers were subjected to thermal oxidation to grow chemically stable silicon dioxide at 1040 °C in the combined mode of a dry–wet–dry oxidation process for 1 h. The resulting film thickness was 300 nm, which is sufficient to serve as a reliable protective mask during the bulk micromachining of the silicon. Conventional photolithography was applied to pattern different sizes of square-shaped non-patterned and meander patterned electrodes of the microcapacitor. 

**Figure 1:**
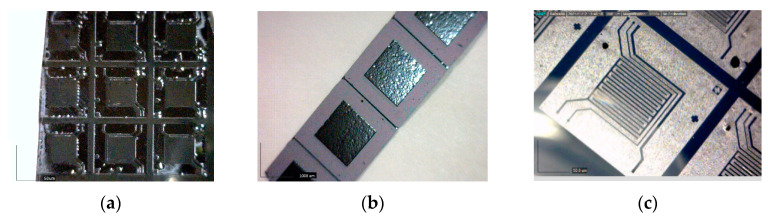

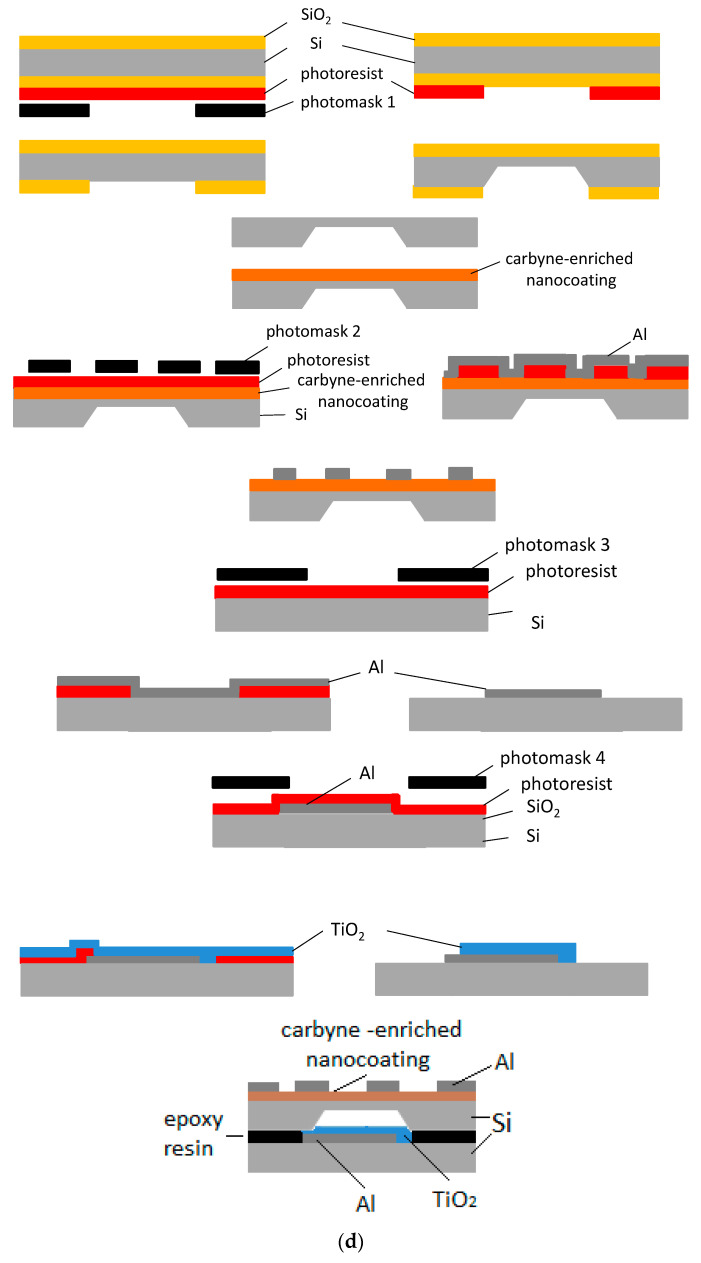

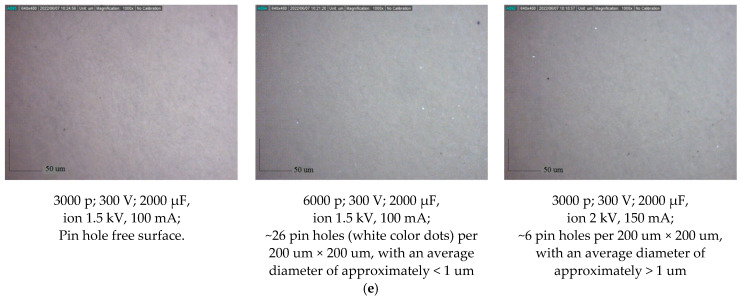
Bulk micromachined silicon membranes: (**a**) small non-patterned membrane with a side size of the square of 65 µm; (**b**) larger non-patterned membrane with a side size of the square of 1000 µm; (**c**) small meander-patterned membrane with a side size of the square of 65 µm; (**d**) technology flow of fabrication of the two parts of the sensing structure and a schematic view of the assembled device; (**e**) optical micrographs of the sensing coatings produced at different deposition conditions.

After the photoresist development, the SiO_2_ protective mask was selectively etched, and then the wafers were immersed in 40% potassium hydroxide solution heated to 60 °C for 4 h to conduct the bulk micromachining. The etching was conducted in a supersonic bath for better uniformity of the cavity profile and reduced roughness of the etched side of the membranes. As a result, flexible membrane of silicon with different sizes (65 µm and 1000 µm of the square side) and geometries (patterned or non-patterned for the smaller membranes) were obtained (Figure 1a–d). The aim of studying different types of the membranes was to find the more appropriate sensitivity and response time, as it is known that these sensors’ characteristics strongly depend on their geometry [12]. Figure 1e represents optical micrographs of the sensing coating, grown at different deposition conditions, namely different number of pulses and different ion plasma powers.

Over the prepared membranes, carbyne-enriched coating was grown as a sensing layer using the ion-assisted pulse-plasma deposition. The main components of the pulse-plasma deposition reactor for growing the 2D-ordered linear-chain carbon nano-matrices are vacuum chamber, pulse-plasma carbon generator, ion source for ionic stimulation, and target assembly with removable target material. The ion and plasma beams intersect above and at the substrate surface. The ion beam irradiation of the deposition zone forms bends in the attached carbon chains, which stabilize the growing chain ensemble. The evaporation of the carbon plasma sheaf from the main discharge graphite cathode is caused by local heating of the graphite surface by electron bombardment to T = 3000 °C. The graphite cathode temperature measurement was provided by a non-contact digital infrared pyrometer. The chains of carbon atoms, C_n_ (where n = 1, 2, 3, …), formed in the plasma sheaf are directed by electrode system to impinge upon the surface of the substrate where the poly-condensation of the carbon chains takes place. The specific energy of the plasma pulse should exceed the breaking energy of the sp^2^ bonds (614 kJ/mol) and the sp^3^ bonds (348 kJ/mol) but should not exceed the breaking energy of the sp^1^ bonds (839 kJ/mol) in the evaporating carbon chains. The controlled bond breaking and sp-phase transformation can be provided through the predictive ion-assisted stimulation with specific energy levels. The optimum deposition conditions were found to be a distance between the target and the substrates of 1 m; 3000 pulses of carbon plasma; voltage for an arc discharge ignited between the main discharge cathode holding the source of carbon and the main discharge anode holding the substrate of 300 V; charge of the main capacitor that formed pulses of carbon plasma of 2000 µf at 5 Hz; and Ar-ion plasma power of 150 W. The thickness of the carbyne-enriched layer was ~50 nm. Previous Raman and X-ray photoelectron spectroscopy study confirmed that the carbyne-like sp^1^-hybridized structures were present in the samples [13,14]. Figure 1e shows that for the highest ion plasma power of 300 W, a few relatively large pinholes (with diameter greater than 1 µm) appeared. For the lower ion plasma power of 150 W and 6000 pulses instead of 3000 pulses, a greater number of smaller pinholes appeared (smaller than 1 µm in diameter). The difference in the number of pin holes was almost 4 times, counted for an area of 200 µm × 200 µm. For the lower ion plasma power of 150 W and lower number of pulses of 3000 instead of 6000 pulses, a pinhole free surface can be observed.

At the final step, the sensing coated micromembranes were assembled to stationary silicon wafers, coated with aluminum electrode, and vacuum-sputtered with dielectric TiO_2_, thus forming metal-oxide-semiconductor (MOS) microcapacitor devices with flexible electrodes and changeable capacitance due to membrane deflection according to the absorbed ethanol molecules and the corresponding membrane geometry. Both silicon pieces were fabricated and processed separately and finally stacked with an epoxy adhesive resin, forming a small cavity in between them. The thickness of the cavities was approximately 205 µm. It is expected that the Van der Waals adsorption process of ethanol molecules will take place on the sensing layer’s surface, which suggests a lack of temperature dependence of the detection mechanism. Microsensors made from silicon are elastic, and the mechanical hysteresis is negligible. In addition, silicon has a low coefficient of thermal expansion, which enables applications in environments with strong variation in the temperature, together with gases, or solvents, without degradation, or parameter changes [15]. Moreover, the dielectric permittivity of the titanium dioxide remains unchanged up to 80 °C [16]. All this is beneficial for the sensor performance, making the device’s response insensitive to the temperature variations. 

For the characterization of sensing devices, a laboratory-made measurement chamber was used. A heater was situated inside the chamber, and on its top, a Petri dish was filled with 96% concentrated liquid ethanol. A current controller with thermocouple feedback set the current through the heater for the precise control of the temperature rate change, thus establishing a specific evaporation rate for the ethanol, converting it into ethanol vapor distributed in the chamber with a certain volume via control of the vaporization time. An additional reference ethanol sensor (MQ-3 (ME075)) with the necessary resolution was mounted close to the device under study. A schematic view of the testing setup can be found elsewhere [17]. The device response was recorded by Impedance analyzer Hioki IM3590 (Hioki E.E., Nagano, Japan). To distinguish the response time of the capacitor from the response time of the sensing layer itself, the full impedance of the sensor device Rs was measured at a constant ethanol concentration of 750 ppm, which was selected because the all three sensors exhibited a linear response around this value. The concentration was changed from 0 to 750 ppm with a uniform rate of vaporization of 150 ppm/s by using a current controller with thermocouple feedback setting the current through a heater for the precise control of the temperature rate change. A Petri dish with a certain quantity of ethanol was put on the heater, thus establishing a specific evaporation rate and converting it into ethanol vapor distributed in the test chamber via the control of the vaporization time. 

## 3. Results and Discussion

**Figure 2:**
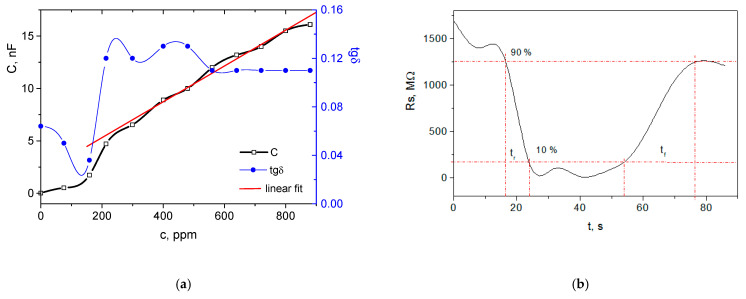
Response of the sensing device with the small meander-patterned membrane: (**a**) capacitance and loss tangent changes with the ethanol concentration; (**b**) sensing layer resistance change with the time for response and recovery time determination at 750 ppm.

Figure 2a shows variation in the device capacitance C and the loss tangent tgδ as a function of the ethanol concentration c in the chamber for the small meander-patterned membrane. The loss tangent factor is the measure of signal loss due to the inherent dissipation of electromagnetic energy in the media, which in this case is the silicon micromembrane coated with carbyne-enriched material. It is the tangent of the angle by which the current in a non-ideal capacitor lags the current in an ideal capacitor. For dielectrics with small loss, this angle is smaller than 1, which is an indication of a capacitive behavior of the overall microdevice. As can be seen from the figure, the device responded in the whole studied range of concentrations from 0.1 to 900 ppm with different sensitivity.

The sensitivity up to 220 ppm was an average of 8 pF/ppm and non-linear, which hinders the direct determination of the concentration. In this range, the adsorbed ethanol molecules are still not sufficient to adhere to the carbyne-enriched nanocoating and establish a stable membrane deflection. The reason could be the limited coverage of the sensing layer over only the meander shaped patterns. This was confirmed by the loss tangent, which in the case of non-polarizable dielectric, reflects only the change in the sensing layer density due to the additional attachment of an analyte. Irregular behavior of the tgδ was observed up to 220 ppm, after which it increased almost three times and was established at an average value of 0.11. Beyond 220 ppm, the sensor response was characterized by a very good linearity, and the average non-linearity was found to be 2%. The maximum capacitance determined by the geometry of the sensor was 16.1 nF, which is relatively small, but the expected value, considering the size of the membrane and the patterned coverage with the sensing layer. Despite the small capacitance, the overall range of the capacitance change was 14.37 nF, 11.4 nF of which belongs to the linear range. Thus, the sensitivity in the linear region was calculated to be 16 pF/ppm. The response time was determined to be 7.9 s, and the recovery time was three times slower (22.4 s, Figure 2b), which can be ascribed to the kinetics of the Van der Waals desorption, which is diffusive, and naturally, it was not thermally activated. The measured values for the sensor response are in the same range of tenths of seconds, as reported in the literature [18,19]. Still, the slightly shorter response time in this paper can be ascribed to the mass-loading sensing principle, which is less inert compared to the change in the dielectric permittivity. In addition, the patterned membrane is lightweight. 

**Figure 3:**
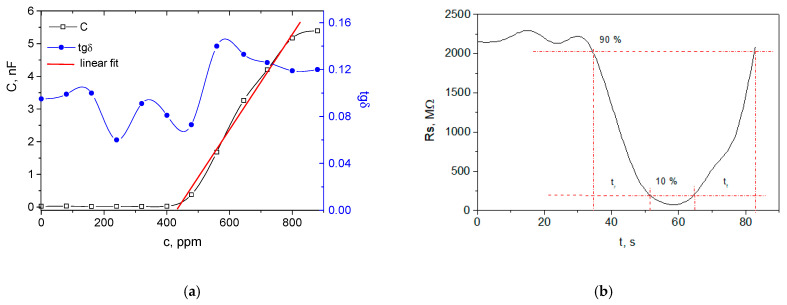
Response of the sensing device with the small non-patterned membrane: (**a**) capacitance and loss tangent changes with the ethanol concentration; (**b**) sensing layer resistance change with the time for response and recovery time determination at 750 ppm.

The next sensor device was designed with the same area of the membrane, but non-patterned, aiming to achieve greater flexible area covered with the carbyne-enriched nanocoating and therefore greater capacitance change. The capacitance change vs. ethanol concentration is shown in Figure 3a. 

The device is almost insensitive before 400 ppm, probably because the mass-weight of the sensing layer itself narrows the dynamic range. This was confirmed by the lower maximum capacitance of 5.39 nF. Thus, a dynamic range between 400 and 900 ppm was obtained, but only the range from 480 to 800 ppm was linear. The average non-linearity was similar to the patterned membrane with the same square size, and it was approximately 2%. The sensitivity in the linear range was estimated to be 15 pF/ppm, which is closer to the case of patterned membrane. The average value of the loss tangent was 0.12, which is an indication of a similar degree of change in the gas-sensing-layer density. Again, it was established at almost constant level in the linear region of detection, which can be ascribed to uniform and stable adsorption of analyte molecules. Because of the higher density of the membrane, the response time was measured to be approximately twice as high (16.9 s) and the recovery time was slightly shorter than the patterned one (18.1 s), probably due to better distribution of the carbyne-enriched nanocoating over the non-patterned surface (Figure 3b). 

**Figure 4:**
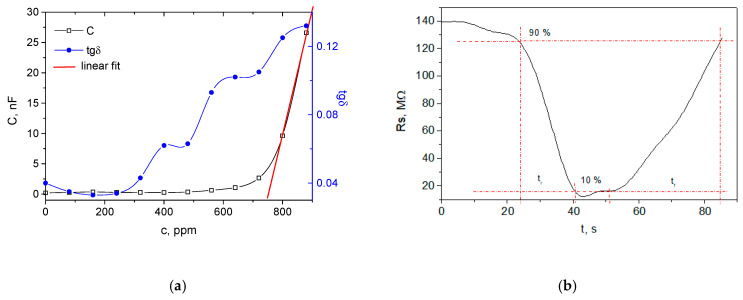
Response of the sensing device with a large non-patterned membrane: (**a**) capacitance and loss tangent changes with the ethanol concentration; (**b**) sensing layer resistance change with the time for response and recovery time determination at 750 ppm.

Figure 4 shows the basic sensor characteristics for the largest membrane with a square side of 1000 µm without patterning. As was expected, following the trends observed for the smaller membranes, the dynamic range of detected concentration is in the reverse direction with the area of the membrane due to the larger quantity of carbyne-enriched material distributed over the flexible electrode of the microcapactor. A range of detected concentration was determined from 480 to 900 ppm, but the response is linear only in the range of 640–900 ppm (Figure 4a). Despite the high mass-weight of the sensing layer, the size of the membrane was much greater compared with the first two studied devices with a 65 µm square size. This is the reason for dominated factor of the electrode area for the higher maximum capacitance of 25.65 nF. The uniform distribution of the sensing layer over the large membrane area resulted in an excellent linearity with an average deviation from the linearity of 1.16%. The sensitivity in the linear region was the greatest from all studied microcapacitors and was estimated to be 98 pF/ppm. The response time was in the same order of magnitude as for the non-patterned small membrane, which is in agreement with the suggestion for the adsorptive mechanism of detection. The recovery time was almost twice as long (34.1 s), which is expected considering the larger area that should be unloaded from the analyte molecules without thermal energy (Figure 4b).

A summary of the values of the basing sensing parameters and characteristics for the three studied devices can be found in the comparative Table 1. As a general trend for all the kinds of sensors studied, it can be highlighted that they are not full recoverable after the removal of the ethanol vapors. As membrane-type silicon capacitors have no hysteresis, this one can be ascribed to the impossibility for full unloading of the adsorbed mass without additional thermal energy. This is in agreement with the suggested interaction mechanism, which is probably a physical adsorption based on Van der Waals forces.

It is expected that the sensing process is not strongly dependent on the ambient humidity and oxygen, because the terminal alkynes on the chain are capped with inert groups (such as tert-butyl or trifluoromethyl) rather than hydrogen atoms [20]. This characteristic feature is related to the growing process, which can cause intrinsic defects due to the reconstruction of the lattice in non-hexagonal rings, such as pentagons, hexagons, and heptagons [21]. Thus, it is likely that such defects, which are intrinsically ascribed to the layer synthesis, together with the edge defects, are responsible for the poor reactivity to relative humidity while enhancing the reactivity to some volatile organic compounds, such as ethanol for example [22,23].

**Figure 5:**
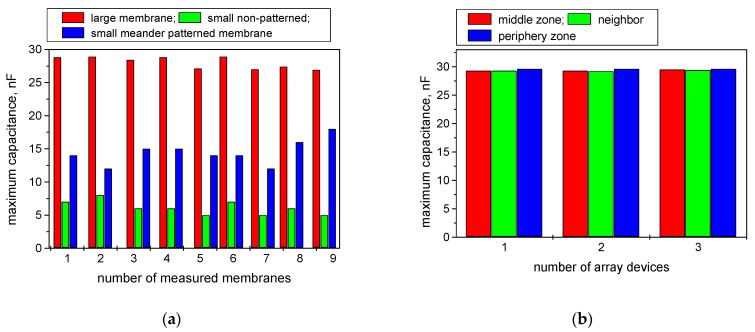
Repeatability data for the membrane-type carbyne-coated sensing structures: (**a**) according to the membrane shape and size; (**b**) according to the sensing element location across the substrate.

Regarding the repeatability of the results, the measurements of nine devices of each type of membranes are provided in Figure 5a. Each device was fabricated as an array from 3, 9, or 12 membranes (according to their size) on a single substrate. They were further simultaneously coated by carbyne-enriched film. The standard deviation for the large membranes was 0.89%, for the small non-patterned was 1.05%, and for the small patterned was 1.87%. The trend of increased dispersion of the values relative to their means with the membrane area decrease can be explained by the fine and complex topology patterns, which probably caused irregularities during the carbyne nanocoating growth. It was expected that the effect of the topology would be the most visible for the most complex meander-shaped membrane. 

Conclusions can also be drawn about the distribution of the measured capacitance according to the location of the membranes in the array from the same substrate, i.e., taken from the middle zone of the array, around the middle zone, and from the periphery. The results suggest good uniformity of the nanocoating across the surface of the array. In addition, the distribution of the capacitance of uncoated membranes was provided, considering the large one, which is expected to have the largest area of the back membrane’s side, which became rough after chemical etching (Figure 5b). The irregular profile may affect the response of the sensor device. The irregularity was estimated by a profilometer, Tencor Alpha Step, for the large membranes and was found to be an average of 840 nm for an area of 1 mm^2^. Very close results were found for three identical arrays that were measured, suggesting that the irregularities of the back side of the membrane are sufficiently small and therefore do not greatly affect the ethanol-sensitivity results. The standard deviation was found to be 0.57% for the middle membranes, 0.67% for neighboring membranes, and 0.94% for the peripheral membranes. The greatest deviations of the peripheral membranes are probably due to edge effects and substrate holder effects, affecting the distribution of the etching solution flow.

## 4. Conclusions

The fabricated sensing silicon-based devices exhibited strong dependencies on the detection parameters of the membrane’s size and patterning surface. It was found that the sensors’ performance was a tradeoff between the sensitivity, dynamic range, and response time. The results present the first steps in demonstrating the controllable response of capacitive sensors based on carbyne-enriched nanocoating to ethanol vapors as a representative of volatile organic compounds. In contrast to other capacitive ethanol sensors, the principle reported in the present paper is mass-sensitive and relies on the deflection of bulk micromachined silicon membranes instead of the sensing layer’s dielectric permittivity change. In this way, the temperature and moisture dependence of the dielectric permittivity was eliminated as an error factor in the measurement. The carbyne-enriched layer discriminates the moisture during the Van der Waals adsorption of the ethanol molecules. 

Future work will be related to study the selectivity of the sensor toward the mixture of similar VOCs. As the full recovery of the initial state of the sensor after exposure to a certain vapor concentration was not observed, the optimization of this parameter will also be a focus of future experiments. It is suggested that faster recovery time and full restoration of the starting impedance can be achieved via the integration of a microheater on the backside of the membrane, facilitating the desorption process. The microheater topology is compliant and aligned with the micromembrane pattern. 

## Figures and Tables

**Table 1:** Main ethanol detection parameters of the capacitive devices with different membrane sizes and patterns, using carbyne-enriched coating as a sensing material.

Parameter/Membrane Type	Linear Dynamic Range, ppm	Range of Capacitance Change, nF	Sensitivity, pF/ppm	Linearity, %	Response Time, s	Recovery Time, s
Small patterned	220–900	14.37	16	2	7.9	22.4
Small non-patterned	480–800	5.37	15	2	16.9	18.1
Large non-patterned	640–900	25.65	98	1.16	16.6	34.1

## Data Availability

Data are available on request due to potential proprietary restrictions.
